# Urbanisation and incidence of acute lymphocytic leukaemia among United States children aged 0–4

**DOI:** 10.1038/sj.bjc.6602607

**Published:** 2005-05-10

**Authors:** A S Adelman, C C McLaughlin, X-C Wu, V W Chen, F D Groves

**Affiliations:** 1 Department of Biostatistics, Bioinformatics and Epidemiology, Medical University of South Carolina, 135 Cannon Street, Charleston, SC 29425, USA; 2 New York State Cancer Registry, Corning Tower, Room 536, Empire State Plaza, Albany, NY 12237, USA; 3 Epidemiology Program, School of Public Health, Louisiana State University Health Sciences Center, 1600 Canal Street, Suite 800, New Orleans, LA 70112, USA; 4 Department of Epidemiology and Clinical Investigation Sciences, School of Public Health and Information Sciences, University of Louisville, 555 South Floyd Street, Room #4053, Louisville, KY 40202, USA

**Keywords:** leukaemia, children, race, urban–rural differences

## Abstract

Acute lymphocytic leukaemia (ALL) incidence among children under 5 years of age was examined, utilising data from 24 United States cancer registries. County-based incidence rates among white children were compared across four levels of urbanisation: large and small metropolitan counties, and adjacent and nonadjacent rural counties. In metropolitan areas, the incidence of ALL was lower among blacks (rate ratio (RR)=0.38, confidence interval (CI)=0.33–0.44) and among Asians/Pacific Islanders (RR=0.78, CI=0.63–0.97) than among whites. Among white children, the incidence of ALL decreased across the four strata of urbanisation, from 67 to 62 to 65 to 54 cases per million person-years at-risk (two-sided trend *P*=0.009), such that rates were significantly lower in the most remote rural counties than in the most populous metropolitan counties (RR=0.80, 95% CI=0.70–0.91).

Acute lymphocytic leukaemia (ALL) is the most common subtype among children. The peak incidence of childhood leukaemia occurs before age 5; almost half of all cancers diagnosed at ages 2 and 3 are leukaemias, and about 80% of all leukaemias occurring before age 10 are ALL. Incidence of childhood ALL in the US is approximately twice as high in whites as in blacks and approximately 20% higher among boys than girls at age<5 years (Smith *et al*, 1999).

Previous studies of the relationship between childhood ALL and demographic variables such as urban/rural status and population density have yielded inconsistent results. Studies in the UK (Alexander *et al*, 1990, 1996; Gilman and Knox, 1998; Dickinson and Parker, 1999) have generally shown higher rates of ALL or non-Hodgkin's lymphoma (NHL) in rural areas. However, studies in Taiwan (Li *et al*, 1998), Australia (McWhirter and Bacon, 1980), Greece (Petridou *et al*, 1997), Sweden (Hjalmars and Gustafsson, 1999), and the United States (Muirhead, 1995) have shown higher incidence of ALL in urban or high-density regions.

The present study is designed to test the hypothesis that incidence of ALL among US children aged 0–4 increases with increasing degree of urbanisation. Acute nonlymphocytic leukaemia (ANLL) and NHL rates were also computed for comparison.

## MATERIALS AND METHODS

County-level incidence data for haematopoietic and lymphoproliferative malignancies among children under 5 years of age during 1995–2000 were obtained from 24 population-based cancer registries (Arizona, Colorado, Connecticut, Atlanta, Idaho, Illinois, Iowa, Kentucky, Louisiana, Michigan, Montana, Nebraska, New Jersey, New Mexico, North Carolina, Oregon, Pennsylvania, Rhode Island, South Carolina, Utah, Washington, West Virginia, Wisconsin, and Wyoming) participating in the National Cancer Institute's Surveillance, Epidemiology, and End Results (SEER) Program or the Centers for Disease Control and Prevention's National Program of Cancer Registries (NPCR). All registries are members of the North American Association of Central Cancer Registries (NAACCR). The registries included in this study are those which consistently meet high standards for quality (including high data completeness, high internal consistency, low case duplication, low fraction of cases where all information comes from death certificates, and low fraction of missing information on race, gender, and age). Each participating registry specifically consented to provide data for this study. These registries covered about 38% of the US population under 5 years of age in 2000. The cancer cases were classified as ALL, ANLL or NHL according to the SEER-modified International Classification of Childhood Cancer (NCI, 2004), as supplied by the SEER^*^Stat software (Surveillance Research Program, 2002). For the purposes of this study, NHL was defined as any lymphoma not explicitly classified as Hodgkin's lymphoma.

Age-specific incidence rates were computed for children aged 0–4 years, stratified by gender, race, and degree of urbanisation. Race was categorised as ‘white’, ‘black’, ‘Asian/Pacific Islander’ (API), and ‘American Indian/Alaska Native’ (AIAN). Information on specific races in the registries’ data was derived from medical records, coded according to standard codes (Hultstrom, 2002), and then grouped into standard race categories in accordance with federal agency standards for the years that the study data were collected (Office of Management and Budget, 1994). The API category includes Chinese, Japanese, Filipino, Hawaiian, Korean, Asian Indian-Pakistani, Vietnamese, Laotian, Hmong, Kampuchean, Thai, Micronesian-NOS, Chamorran, Guamanian-NOS (not otherwise specified), Polynesian-NOS, Tahitian, Samoan, Tongan, Melanesian-NOS, Fiji Islander, New Guinean, Other Asian, Pacific Islander-NOS, and Oriental-NOS. All API subcategories were combined into one group because population data for the individual subgroups were not available.

Degree of urbanisation was originally categorised according to the 10 levels (0–9) of the ‘rural–urban continuum code’ developed by the United States Department of Agriculture (Beale, 2003a). Counties were first designated as ‘metropolitan’ or ‘nonmetropolitan’. Metropolitan counties were further stratified by population size (greater than or less than 1 000 000 people). The nonmetropolitan counties were divided into subgroups based on adjacency to metropolitan areas as well as percentage of population residing in urban areas. None of the previous studies referenced in this paper (McWhirter and Bacon, 1980; Alexander *et al*, 1990, 1996; Petridou *et al*, 1997; Gilman and Knox, 1998; Li *et al*, 1998; Dickinson and Parker, 1999) dealt with the size of populations, but rather with urban/rural status and distance from urban areas. Therefore, for comparability to previously published studies and to obtain reasonably precise point estimates of incidence rates, we have opted to emphasise the proximity of nonmetropolitan counties to metropolitan areas while collapsing the subcategories of urban population among the nonmetropolitan counties. Counties in this study were grouped into four strata in order of decreasing urbanisation as shown in Table 1. Although individual counties may contain both urban and rural populations, strata 1 and 2 comprise metropolitan counties that are predominantly urban, while strata 3 and 4 comprise nonmetropolitan counties that are predominantly rural.

Cells with fewer than 20 cases are not displayed in order to protect the privacy of the cases, and because when the numbers of cases used to compute incidence rates are small, the reliability of the rates tends to be poor (Brillinger, 1986). Rural–urban gradients over all four strata were assessed using the Cochran–Armitage test for trend (Agresti, 1990). Pairwise rate ratios (RRs) and their confidence intervals (CIs) (for comparing incidence rates by gender, race, and metropolitan/nonmetropolitan status) were calculated using the maximum likelihood estimate (Rothman and Greenland, 1998).

Population attributable fractions due to urbanisation were calculated, taking into account that ‘exposure’ to urbanicity is polytomous and basing the structure of the population attributable fraction on the distribution of exposure in cases (Hanley, 2001).

## RESULTS

A total of 2423 cases of ALL were observed in 42 306 667 person-years at risk in the 24 participating registries. Of the ALL cases, 1921 resided in metropolitan counties and 502 in nonmetropolitan counties. There were a total of 429 cases of ANLL and 144 cases of NHL. Racial characteristics of cases are shown in Table 2.

Incidence rates for ALL, ANLL, and NHL among whites, blacks, and APIs subjects, who resided in metropolitan counties, are shown in Table 3 (data for AIANs are too sparse to be displayed). Black subjects had lower incidence of ALL (RR=0.38, 95% CI=0.33–0.44), ANLL (RR=0.74, 95% CI=0.57–0.95), and NHL (RR=0.56, 95% CI=0.40–0.80) than white subjects, and APIs likewise had lower incidence of ALL than whites (RR=0.78, 95% CI=0.63–0.97). Regardless of race, boys had a higher incidence of ALL (RR=1.17, 95% CI=1.09–1.25, data not shown) and NHL (RR=1.99, 95% CI=1.57–2.52, data not shown) than girls, while ANLL rates did not vary by gender (RR=1.09, 95% CI=0.93–1.29, data not shown).

Due to sparse data for non-whites, the rural—urban gradients were assessed using only the data for whites (Table 4). There were no rural–urban gradients in incidence of ANLL or NHL among whites of either gender. Among whites of both genders, the incidence of ALL decreased across the four strata of urbanisation, from 67–62 to 65–54 cases per million person-years at-risk (two-sided trend *P*=0.009), such that rates were significantly lower in the most remote rural counties than in the most populous metropolitan counties (RR=0.80, 95% CI=0.70–0.91). The rural–urban gradient was even stronger among boys (RR=0.71, 95% CI=0.59–0.85, two-sided trend *P*=0.008), but was not statistically significant among girls (RR=0.92, 95% CI=0.76–1.10, two-sided trend *P*=0.31), such that the overall rural–urban gradient for children of both genders was driven by the gradient among boys (data not shown).

The proportion of childhood ALL cases attributable to any degree of urbanisation beyond ‘rural, nonadjacent’ was 16% (24% for boys *vs* 5% for girls, data not shown).

## DISCUSSION

We found that incidence of ALL among white children was higher in urban areas than rural areas. Among white boys aged 0–4 years in rural areas, it was 29% lower than among their urban counterparts; however, no such rural–urban gradient was detected among white girls.

Previous studies in Australia, Greece, Taiwan, Sweden, and the United States also found increased rates of childhood ALL in urban areas. Acute lymphocytic leukaemia incidence among children living in Brisbane City, Australia, during 1973–1979 was significantly higher than among children living in the (more rural) remainder of the Brisbane Statistical Division (RR=1.59 for children 0–4 years old) (McWhirter and Bacon, 1980). In Greece during 1980–1989, childhood ALL incidence was higher in urban areas than in semiurban or rural areas (Petridou *et al*, 1997). Similarly, leukaemia incidence rates among children aged 0–4 residing in the 361 districts of Taiwan increased monotonically across four levels of urbanisation, with RRs of 1.0, 1.2, 1.3, and 1.5 in ‘rural areas’, ‘towns’, ‘cities’, and ‘metropolitan areas’ (trend *P*<0.01) during 1981–1990 (Li *et al*, 1998). The RRs for whites between the extremes in urban status in our study (1.2 for both ALL and ANLL) were comparable to those reported by Li.

Some researchers have assessed the effect of population density, as well as urbanisation *per se*, on incidence of ALL. Incidence of ALL among Swedish children under age 15 during 1973–1994 (Hjalmars and Gustafsson, 1999) was significantly higher in ‘population centres’ than in the Swedish countryside (3.99 *vs* 2.38 cases per 100 000; OR=1.68; 95% CI=1.44–1.95); no significant rural–urban gradient was observed for ANLL (0.67 *vs* 0.59 cases per 100 000; OR=1.13; 95% CI=0.98–1.32). The childhood ALL incidence rates in Sweden increased monotonically with increasing degrees of urbanisation (3.32, 3.41, and 3.65 cases per 100 000 in ‘rural’, ‘semiurban’, and ‘urban’ areas, respectively), and also with increasing population density (3.55, 3.63, and 4.09 cases per 100 000 in areas with <400, 400–800, and >800 persons per square kilometer, respectively). Likewise, white children under 15 years of age during 1978–1982 in high-density census tracts in three US metropolitan areas (San Francisco-Oakland, Detroit, and Atlanta) had higher combined incidence rates of leukaemia and NHL (RR=1.4) than those in low-density census tracts. Borderline elevations in incidence of leukaemia and NHL were detected among whites in medium-density areas (RR=1.3) and among all races in medium-density (RR=1.2) and high-density (RR=1.3) areas (Muirhead, 1995). Since Muirhead dealt with population density only within urban areas, his RRs are not directly comparable to ours. Our study builds upon Muirhead's analysis by incorporating more recent data (1995–2000 instead of 1978–1982) from many more cancer registries and by directly comparing ALL incidence in urban *vs* rural counties instead of just in urban counties.

In contrast with previously published findings in Taiwan (Li *et al*, 1998), Australia (McWhirter and Bacon, 1980), Greece (Petridou *et al*, 1997), Sweden (Hjalmars and Gustafsson, 1999), and the United States (Muirhead, 1995), studies in the United Kingdom found higher incidence of childhood ALL in rural areas. The incidence of ALL among children aged 1–7 years in 3270 electoral wards in England and Wales during 1984–1988 increased over four levels of distance from built-up areas (RR=2.2; trend *P*=0.002) for wards >20 km from a built-up area (Alexander *et al*, 1990, 1996). Likewise, in a multivariate study of cancer among a cohort of children aged 1–14 born in the rural English district of Cumbria during 1969–1989, those who did not reside in a built-up area had an RR for ALL/NHL of 2.0 compared with those who resided in a built-up area (Dickinson and Parker, 1999). Paediatric (ages 0–15) leukaemia and lymphoma mortality (adjusted for birth density, birth year, and radon exposure) in inner London during 1953–1980 was significantly lower than in less-urban areas in the UK (RR=0.80) (Gilman and Knox, 1998). However, the differences in mortality may have reflected higher survival rates in London than in outlying areas.

Our finding of a rural–urban gradient in ALL incidence among boys but not girls, while unexpected, was not unprecedented. There was a male predominance of ALL among Greek children in urban areas (65.9 per million boys *vs* 56.5 per million girls) and semiurban areas (45.3 per million boys *vs* 42.5 per million girls), although the opposite was true in rural areas (42.2 per million boys *vs* 46.4 per million girls) (Petridou *et al*, 1997). Furthermore, there was greater geographic variation in ALL incidence among white boys than among white girls, as reported by 12 US cancer registries during 1973–1986 (Linet and Devesa, 1991).

It must also be noted in our study that just as there is no rural–urban gradient in ALL incidence among girls, there is likewise no gender disparity in ALL incidence in the most rural areas. Thus, one could argue that exposure to some unknown urban-associated risk factor occurs more commonly among boys than among girls. Alternatively, white boys in urban areas could be more susceptible to an unknown risk factor.

This is an ecologic study; it does not deal with individual subjects or individual-level traits or exposures, but rather with the characteristics of large groups of counties. As such, it is possible that positive results among white boys may be due to genetic or environmental risk factors associated with urban residence in the United States that were not assessed in this study. It is also impossible to determine from our data whether or not cases developed cancer in the same counties where they were diagnosed; conceivably, children with preclinical cancer could be more likely to move from rural to urban areas than *vice versa*.

It is also conceivable that the considerable difference in county size east and west of the Rocky Mountains could affect ecologic measures (e.g. small counties would be expected to be more homogeneous than large counties), but since we have lumped Beale's 10 continuum codes into a mere four so that population is only a distinguishing factor between the two metropolitan strata, urban/rural composition of large counties probably does not affect this potential problem. That large nonmetropolitan counties, with their bigger perimeters, may be more likely to be adjacent to metropolitan areas than small counties might be more of a concern; however, according to the map of the 1993 version of the codes (Beale, 2003b), there are numerous rural nonadjacent counties in the areas around and on both sides of the Rocky Mountains, so this does not seem to be an actual problem.

Our findings do not conclusively prove that there is an association between urbanisation and childhood ALL, nor have we proven that any risk factor for childhood ALL occurs more commonly in urban areas. Rather, our results indicate that further research into the nature and causes of the rural–urban gradient in childhood ALL is warranted.

## Figures and Tables

**Table 1:** Classification of counties by degree of urbanisation

**Stratum grouping**	**Stratum**	**Rural–urban continuum code^a^**
Urban	1. Counties in metropolitan areas with population ⩾1 000 000	0: ‘Central counties’
		1: Fringe counties
	2. Counties in metropolitan areas with population <1 000 000	2: Population 250 000–1 000 000
		3: Population <250 000

Rural	3. Counties adjacent to metropolitan area	4: Urban population ⩾20 000
		6: Urban population 2500–19 999
		8: Completely rural or urban population <2500
	4. Counties not adjacent to metropolitan area	5: Urban population ⩾20 000
		7: Urban population 2500–19 999
		9: Completely rural or urban population <2500

a Beale C (2003). Description of the rural–urban continuum codes prior to 2003. USDA Economic Research Service: http://www.ers.usda.gov/Briefing/Rurality/RuralurbCon/priordescription.htm.

**Table 2:** Racial characteristics of cases

	**Whites (%)**	**Blacks (%)**	**APIs (%)**	**AIANs (%)**	**Unknown race (%)**
ALL, all counties (2423 cases)	89	7	3	1	1
ALL, metro (1921 cases)	88	8	3	1	1
ALL, nonmetro (502 cases)	92	4	2	1	1
ANLL, all counties (429 cases)	81	13	5	0	0
NHL, all counties (144 cases)	84	10	6	0	0

*Note*: Percentages are given due to privacy concerns associated with small, exact numbers in some categories. For purposes of this study, ALL (acute lymphocytic leukaemia) is defined as *International Classification of Diseases for Oncology, 2nd edn* (ICD-O-2) morphology codes 9821 and 9828; ANLL (acute nonlymphocytic leukaemia) is defined as codes 9840, 9841, 9861, 9864, 9866, 9867, 9871–9874, 9891, 9894, and 9910; and NHL (non-Hodgkin's lymphoma) is defined as codes 9590, 9591–9595, 9670–9688, 9690–9717, 9720, 9723, and 9731–9764.

API=Asian/Pacific Islander, AIAN=American Indian/Alaska Native.

**Table 3:** Paediatric (ages 0–4) cancer incidence rates (per 1 000 000) for both genders combined by race and RRs contrasting incidence rates by race in metropolitan counties of 23 US states and Atlanta, 1995–2000

		**ALL**			**ANLL**			**NHL**		
**Race**	**Person-years at risk**	**No. of cases**	**Rate (95% CI)**	**RR (95% CI)**	**No. of cases**	**Rate (95% CI)**	**RR (95% CI)**	**No. of cases**	**Rate (95% CI)**	**RR (95% CI)**
Black	5 985 970	149	25 (21–29)	0.38 (0.33–0.44)	47	8 (6–10)	0.74 (0.57–0.95)	25	4 (3–6)	0.56 (0.40–0.80)
API	1 167 614	59	50 (38–65)	0.78 (0.63–0.97)	<20	a	a	<20	a	a
White	25 941 007	1685	65 (62–68)	1.00 (Referent)	277	11 (10–12)	1.00 (Referent)	192	7 (6–8)	1.00 (Referent)

*Source*: North American Association of Central Cancer Registries.

a Not shown due to sparse data (*N*<20).

RR=rate ratio, ALL=acute lymphocytic leukaemia, ANLL=acute nonlymphocytic leukaemia, NHL=non-Hodgkin's lymphoma, API=Asian/Pacific Islander, AIAN=American Indian/Alaska Native.

**Table 4:** Paediatric (ages 0–4) cancer incidence rates (per 1 000 000) for both genders combined by degree of urbanisation and gender and RRs contrasting incidence rates by degree of urbanisation in 23 US states and Atlanta among whites, 1995–2000

		**ALL**			**ANLL**			**NHL**		
**Degree of urbanisation**	**Person-years at risk**	**No. of cases**	**Rate (95% CI)**	**RR (95% CI)**	**No. of cases**	**Rate (95% CI)**	**RR (95% CI)**	**No. of cases**	**Rate (95% CI)**	**RR (95% CI)**
Metro⩾1M	14 853 994	997	67 (63–71)	1.00 (Referent)	157	11 (9–12)	1.00 (Referent)	115	8 (6–9)	1.00 (Referent)
Metro<1M	11 087 013	688	62 (58–67)	0.92 (0.85–1.00)	120	11 (9–13)	1.02 (0.84–1.25)	77	7 (6–9)	0.90 (0.70–1.14)
Nonmetro adj.	4 064 046	263	65 (57–73)	0.96 (0.86–1.08)	40	10 (7–13)	0.93 (0.70–1.25)	23	6 (4–8)	0.73 (0.50–1.06)
Nonmetro non-adj.	3 702 524	199	54 (46–62)	0.80 (0.70–0.91)	32	9 (6–12)	0.82 (0.59–1.12)	<20	a	a
		Two-sided trend *P*=0.0090 Pop. attributable fraction=0.15								

*Source*: North American Association of Central Cancer Registries.

RR=rate ratio, ALL=acute lymphocytic leukaemia, ANLL=acute nonlymphocytic leukaemia, NHL=non-Hodgkin's lymphoma.

a Not shown due to sparse data (*N*<20).
